# HELP@APP: development and evaluation of a self-help app for traumatized Syrian refugees in Germany – a study protocol of a randomized controlled trial

**DOI:** 10.1186/s12888-019-2110-y

**Published:** 2019-04-30

**Authors:** Johannes Golchert, Susanne Roehr, Franziska Berg, Thomas Grochtdreis, Rahel Hoffmann, Franziska Jung, Michaela Nagl, Anna Plexnies, Anna Renner, Hans-Helmut König, Anette Kersting, Steffi G. Riedel-Heller

**Affiliations:** 1 Institute of Social Medicine, Occupational Health and Public Health (ISAP), Medical Faculty, University of Leipzig, Philipp-Rosenthal-Straße 55, 04103 Leipzig, Germany; 2 Department of Health Economics and Health Services Research, Hamburg Center for Health Economics, University Medical Center Hamburg-Eppendorf, Hamburg, Germany; 3 Department of Psychosomatic Medicine and Psychotherapy, University Medical Center Leipzig, Leipzig, Germany

**Keywords:** Posttraumatic stress, Trauma, Traumatization, Self-management program, App, mHealth, Digital health, Syria, Refugee, RCT, Intervention

## Abstract

**Background:**

Syrians represent the largest group among refugees in Germany. Many of them were exposed to sequential traumatizing events including war, escape and post-migration stressors, which significantly increase the risk to develop symptoms of posttraumatic stress and other mental disorders. However, there is a lack of adequate treatment options for traumatized refugees in Germany. Moreover, their access to psychosocial care is often restricted due to legal regulation, language barriers, and unclear cost coverage. We therefore aim to develop a low-threshold supportive self-help app for Syrian refugees with posttraumatic stress symptoms. By conducting a randomized controlled trial, we further aim to evaluate the apps’ efficacy, usability, acceptance, and economic health benefit/cost-effectiveness.

**Methods:**

We will develop a modular, interactive self-help app in Arabic, which will be grounded on cognitive-behavioral models for the treatment of posttraumatic stress. Subsequently, screened positive (i.e., Syrian refugees, 18–65 years old, mild to moderate posttraumatic stress symptomatology as quantified by the Posttraumatic Stress Diagnostic Scale for DSM-5/PDS-5) participants (ideally up to *n* = 234) will be randomly allocated to an intervention (IG) and control group (CG), respectively. Participants in the IG will gain access to the self-help app for one month, while participants in the CG will receive psychoeducational reading material in form of a comprehensive brochure on traumatization and posttraumatic stress. Measurements are scheduled before the intervention (T0), directly after the intervention (T1, one month later) and three months after the intervention (T2). Using linear mixed effect models, we will investigate change in posttraumatic symptomatology. We will also test for changes in secondary outcomes such as depression, anxiety, and quality of life. Moreover, we will inspect the usability and user acceptance of the app. To evaluate the app in terms of its economic health benefit, the incremental cost-effectiveness ratio will be calculated.

**Discussion:**

We plan to make the app freely available to the general public after evaluation. Thus, the app can help to add-on to routine care, which currently lacks sufficient and appropriate treatment options for Syrian refugees.

**Trial registration:**

German Clinical Trials Register/Deutsches Register Klinischer Studien (DRKS). Registration ID: DRKS00013782. Registered: 06th of July 2018.

## Background

More than 5.6 million Syrians have fled their home country since the beginning of the Syrian civil war in 2011 [1]. Syrians represent the largest group among refugees in Germany. According to the Federal Office for Migration and Refugees [2], a total number of 158,657 Syrian refugees have applied for asylum in Germany in 2015. Compared to 2014, this corresponded to an increase of approximately 300%. The upward trend continued through 2016, in which another 266,250 Syrians applied for asylum. Including the number of applications from 2017 (48,974) and 2018 (as of November: 41,345), this equals to a total number of 515,226 Syrian refugees in Germany between 2015 and November 2018.

Most of the Syrian refugees were typically exposed to a variety of potentially traumatizing situations. First, they might have been conflicted with wartime events in Syria, including bombardments and other combat operation. Second, the escape itself, which may last for months, sometimes even years (i.e., if they are aiming for European countries), may have caused existential threat, e.g. through dangerous crossings such as the Mediterranean Sea. Third, upon arrival at a host state, refugees are often confronted with so called post-migration stressors, such as discrimination and even physical violence [3]. Moreover, in Germany, refugees have to deal with time-consuming asylum procedures, prolonged waiting periods, and an ambiguous residence status, which can additionally increase the risk for developing psychiatric disorders [3, 4]. Formal hearings as part of the asylum procedure, in which the reasons for the escape are discussed, can be a further post-migration stressor. Indeed, current evidence suggests an increase in the development of intrusive experiences caused by asylum interviews as part of the asylum procedure [5]. Taken together, these events significantly increase the risk for developing trauma related and other disorders [6].

The most frequently reported disorders associated with war traumatization are posttraumatic stress disorder (PTSD) and major depression: studies reported prevalence rates of 33.5% for PTSD [7], and 29.5% for depression [8] among Syrian refugees. By contrast, in a representative study of the European general population that included six countries, the 12-month prevalence rates for PTSD and depression were 0.9 and 3.9%, respectively [9]. PTSD is often accompanied by comorbid physical complaints, of which chronic pain is most common. Among refugees with PTSD, prevalence rates of clinically relevant chronic pain ranged from 76% [10] to 88% [11].

Recent PTSD guidelines by the German Association of the Scientific Medical Societies (AWMF) indicate that a trauma adaptive psychotherapy should be provided for every patient diagnosed with PTSD [12]. During the first 15 months after arrival to Germany, however, psychosocial care for refugees cannot be fully ensured due to initially restricted access to the health care system, which is regulated through specific asylum law (Asylbewerberleistungsgesetz/AsylbLG). By contrast, refugees who have stayed in Germany for more than 15 months gain access to the statutory health insurance that covers psychotherapy. According to a verdict of the Federal Social Court, however, health insurance funds are not obliged to cover the costs for a qualified translator, if needed. Instead, regional/local social security offices make individual decisions whether translator expenses can be refunded. However, refunding cannot be guaranteed, and it can take an enormous amount of extra time until a decision is made [3]. Moreover, the number of specialized psychotherapists and translators is limited. It is thus difficult to provide treatment for PTSD to all refugees in need. Indeed, there is a clear lack of adequate health services in this regard. Likewise, research has shown that traumatized individuals perceive additional barriers (e.g. concerns related to stigma, shame and rejection), which may hinder them to seek for adequate treatment [13].

Computerized/web-based and smartphone-based (apps) self-management programs that address posttraumatic stress in refugees are thus of particular importance as they a) may serve as a first supportive intervention and add-on to routine care, b) could fill a treatment gap in Germany, and c) may lower the threshold for refugees to seek help. Compared to face-to-face interventions, such digital approaches offer anonymity, which can help individuals to take up a respective treatment, who would otherwise maybe refuse to do so, e.g. due to concerns about stigmatization or feelings of shame [14, 15]. With the rapid increase of mobile phone use in recent years, apps for health care have also been developed. Since smartphones serve as an important communication tool and sometimes represent the only possibility for refugees to have access to the internet [16], smartphones might be of particular value in terms of health-related self-care programs in this particular group. As opposed to web-based approaches, mobile health (mHealth)-apps can be used flexibly and independently of internet access. Therefore, they are almost available without restrictions. In addition, mHealth-apps allow real-time monitoring of symptoms and behavior. Moreover, due to the easy and constant accessibility, they might improve treatment adherence [17]. Evidence for the efficacy of mHealth-apps has been reported for depression, anxiety, stress, and substance abuse [17, 18]. Likewise, a smartphone-based app for posttraumatic stress has proven to be efficient in veterans and in a general adult sample [19]. Two recent meta-analyses of internet-based interventions for posttraumatic stress reported medium to large effect-sizes with respect to a decrease in symptom severity [20, 21]. Thus, such interventions have proven to be effective [22].

### Objectives

Taken together, Syrian refugees are exposed to a variety of traumatizing situations making them especially vulnerable to posttraumatic stress symptoms. In Germany, psychosocial/psychotherapeutic care for refugees, however, is restricted by legal regulations and often unclear cost coverage. Moreover, there is a lack of easily accessible language and culture-sensitive interventions for posttraumatic stress in refugees. Therefore, we aim to develop an app-based self-management program in Arabic language for traumatized Syrian refugees in Germany. We further aim to evaluate the apps’ efficacy and cost-effectiveness by conducting a randomized controlled trial (RCT) with two arms. Participants in the intervention group (IG) will gain access to the self-help app. Participants in the control group (CG) will be provided comprehensive psychoeducational reading material regarding PTSD. Using linear mixed effect models, we will investigate change in posttraumatic symptom load and related outcomes, such as depression, anxiety, and quality of life. App usability and user acceptance will additionally be assessed. To evaluate the app in terms of its health economic benefits, the incremental cost-effectiveness ratio (ICER) will be calculated.

### Expected benefits and harms

We expect that the low-threshold app-based intervention will lower posttraumatic symptom severity and associated secondary outcomes in favor of overall improved wellbeing and quality of life. Furthermore, since our self-help app will be in Arabic language, basal information regarding treatment options and disease management can be provided to a broad group of traumatized refugees without causing costs due to translation. This can help to compensate for the lack of adequate psychotherapeutic care for Syrian refugees in Germany. In case of an unexpected increase in symptom severity - the trauma-related content might initially trigger unpleasant memories and feelings - both study groups will be provided with contact information for professional help in case of emergency. Moreover, the occurrence of negative effects, regardless whether or not related to the trial, will be monitored.

## Methods

We aim to develop and evaluate a self-help app for traumatized Syrian refugees. This study protocol describes both steps in detail in adherence to the SPIRIT 2013 statement (Standard Protocol Items: Recommendations for Interventional Trials) [23].

### Development of the self-help app

#### Theoretical and clinical framework

Our interactive self-help app will rely on (i) evidence-based cognitive-behavioral therapy (CBT) for PTSD [24], and (ii) evaluated internet-based self-care approaches for the treatment of posttraumatic stress [25, 26]. Critically, meeting the specific needs of refugees as well as adapting the content in a culturally sensitive manner will be key to the development of our self-help app. Initially, interviews with approximately 5–10 target persons will thus be carried out to assess culturally sensitive aspects (e.g. different concepts of disease and disease management), that may be important for the intervention. To ensure full functionality and usability of the app, representatives of the target group will accompany all steps of the developmental process through regular consultation.

#### Structure and content of the self-help app

**Fig. 1:**
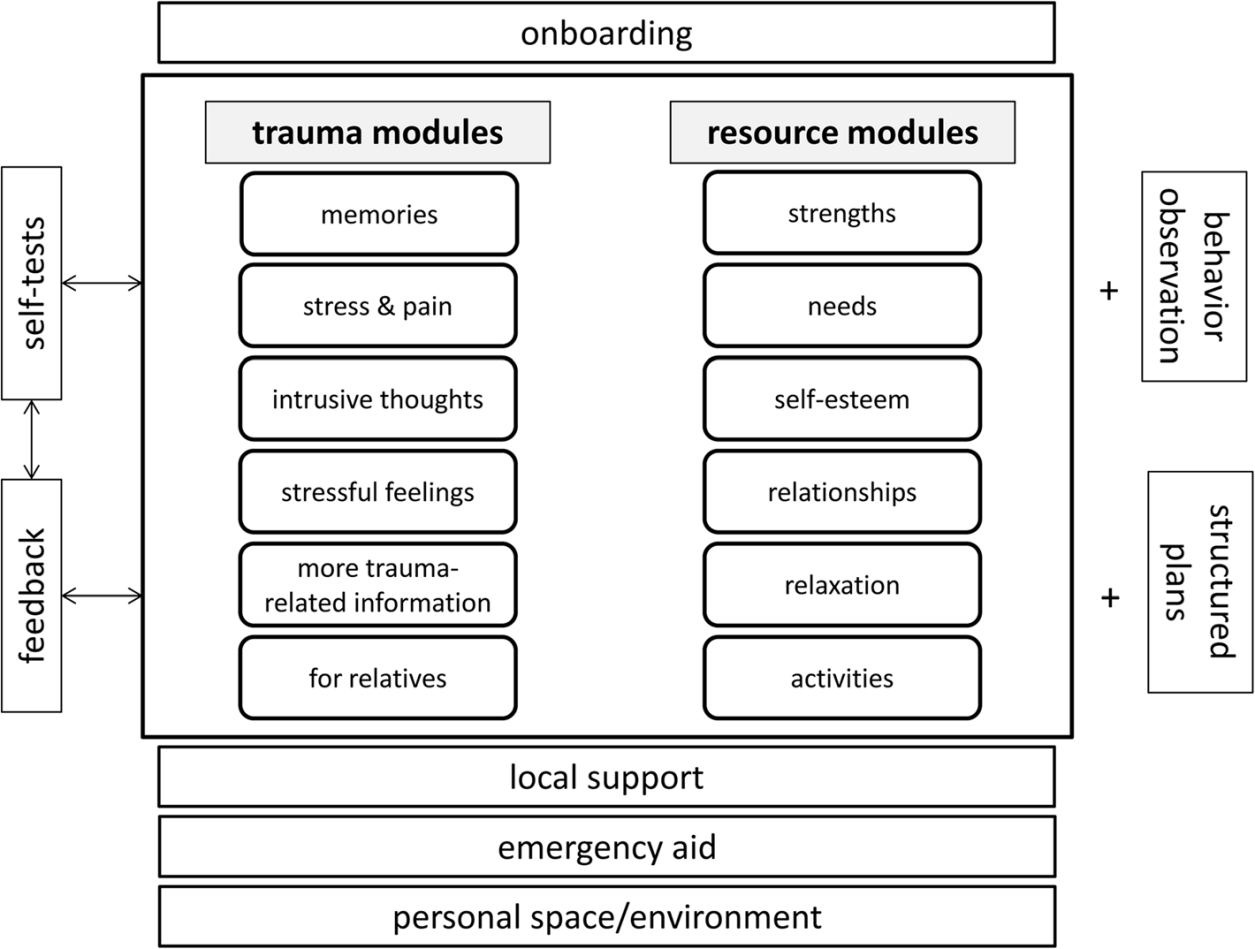
Study design for a randomized controlled trial to evaluate a self-help app for traumatized Syrian refugees living in Germany

As can be seen in Fig. 1, the app will be set up modularly. Initially, participants will be guided through general user instructions and information about the association between war, escape, and posttraumatic stress will be provided (“*onboarding”*). Then, participants will be able to flexibly work through two modules (“trauma” and “resources”), which will consist of several submodules.

The “trauma”-submodules will address consequences of traumatization, especially focusing on posttraumatic stress symptoms as defined by the *Diagnostic and Statistical Manual of Mental Disorders, 5th Edition* (DSM-5) [27]. In addition, symptoms of depression and anxiety will be introduced and explained. All submodules will provide (i) *psychoeducational information* (e.g. what are intrusions and flashbacks, what is somatization), and (ii) basic *self-help techniques and skills* with respect to symptom management (e.g. positive and negative coping strategies, cognitive restructuring, emotion regulation). We will also implement a self-test (PTSD Checklist for DSM-5/PCL-5) [28], that can be completed at any time in order to monitor symptom severity and to provide automated individually tailored feedback regarding progress and potential problems.

The “resources”-submodules will impart (i) *psychoeducational information* (e.g. about physical and mental needs, consequences of experienced traumata on self-esteem and relationships) as well as (ii) *techniques and skills* to strengthen and support own resources and self-care (e.g. setting goals, planning pleasant activities, gaining social support, relaxation techniques, mindfulness).

#### Usability

To maximize usability, content related materials will be provided in Arabic. Moreover, the app will additionally be complemented by video and audio sequences as well as interactive games and exercises. All submodules will be available at all times, giving users the possibility to flexibly switch between sections depending on their personal needs. The app will also provide a clearly visible emergency button with contact information regarding professional help in case of emergency. The app will be usable offline.

With respect to the technical implementation, the app development will take place in cooperation with the e-mental health program provider “Frühlingsproduktionen”.

### Evaluation of the self-help app

#### Participants

Study participants will be Syrian refugees living in the urban area of Leipzig, Germany, who will be recruited using a multi-strategic approach [29]. If contacted refugees are willing to participate, written informed consent will be obtained. Trained native Arabic speaking study nurses will screen up to *n* = 1200 refugees for eligibility defined by the following inclusion and exclusion criteria, respectively.

*Inclusion criteria*:

- Syrian refugee living in Leipzig, Germany, aged 18–65 years
- experience of at least one traumatic event and subsequent mild to moderate posttraumatic stress symptom severity (Posttraumatic Diagnostic Scale for DSM-5/PDS-5 = 11–59 [30])
- owning a compatible device (Android / iOS)

*Exclusion criteria:*

- severe posttraumatic stress symptomatology (PDS-5 ≥ 60 [30])
- severe depressive symptomatology (Patient Health Questionnaire/PHQ-9 ≥ 20 [31])
- acute suicidal tendencies (Depressive Symptom Inventory-Suicidality Subscale/DSI-SS ≥ 3 [32])
- current psychotherapy/psychiatric treatment and/or psychopharmaceutical medication
- pregnancy

#### Intervention

**Fig. 2:**
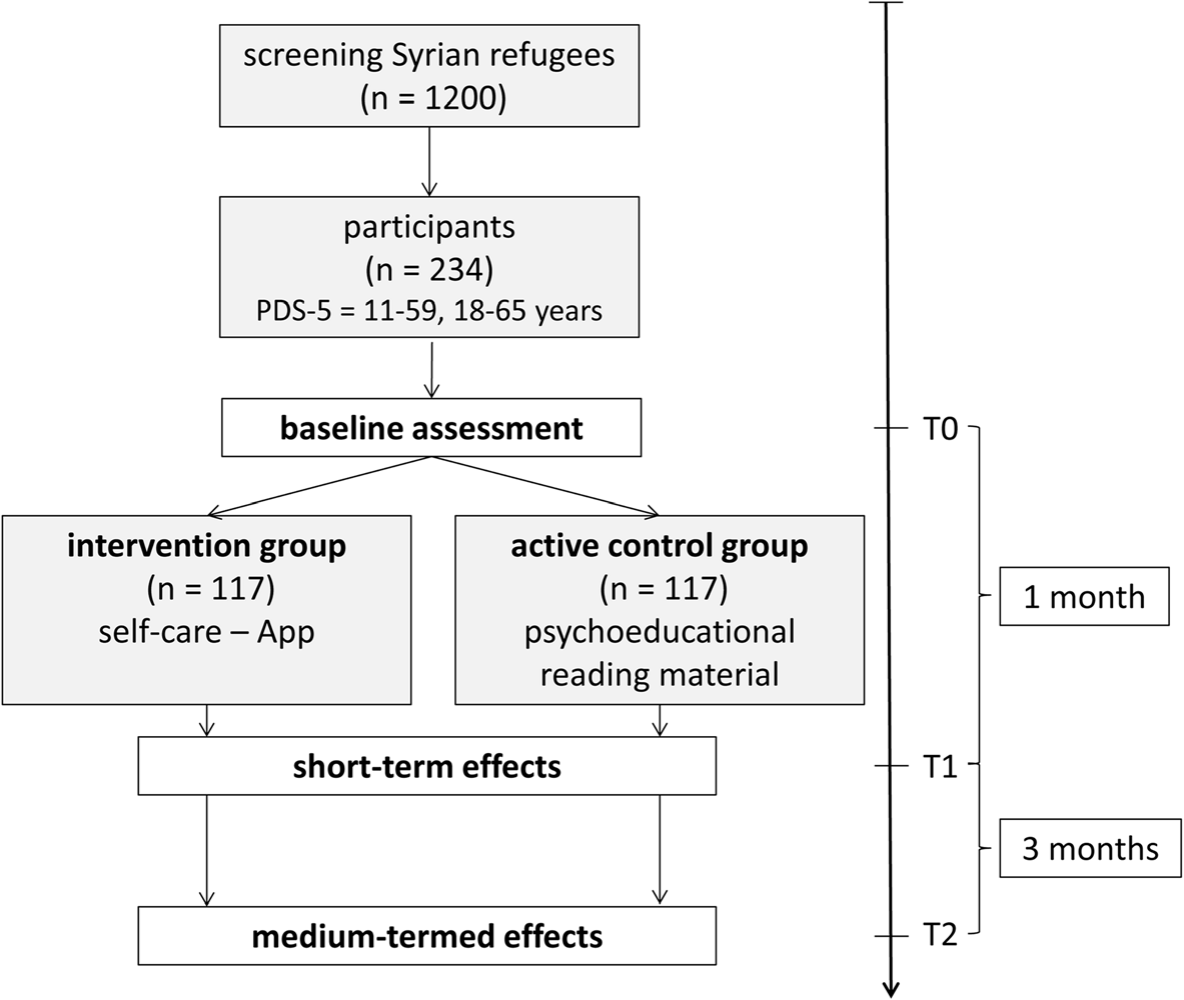
Overview of the structure and contents of a self-help app for traumatized Syrian refugees living in Germany

The apps’ efficacy and usability as well as the overall associated cost effectiveness of the intervention will be evaluated in a prospective RCT with an IG and a CG (see Fig. 2). Following the initial screening and study group assignment, participants in the IG will gain access to the self-help app via person-specific log-in data (to avoid group contamination) for one month. During that time, the CG will obtain psychoeducational reading material about traumatization and posttraumatic stress (identical to the information delivered by the app) in form of a comprehensive brochure in Arabic.

#### Study adherence

Prior to randomization, participants will be pointed towards the required investment of time and effort necessary to complete the study in order to lower the risk for dropout.

Treatment adherence of the IG will be objectively assessed through the apps’ meta-data log-files that store information about the number of login-times and worked-through modules. Likewise, dropout assessment will be conducted to understand reasons for non-adherence.

Participants of both groups are assured to contact the study nurses in case of arising problems regarding current symptom burden, for example. Similar to the apps’ emergency button, the psychoeducational reading material will clearly state contact information regarding professional help in case of emergency.

#### Outcomes

Table 1 gives an overview of all outcomes and associated measures to be implemented throughout the entire study. Instruments that are not available in Arabic language will be translated according to recommendations of the “European Social Survey Programme” and “Cross-Cultural Survey Guidelines” respectively [33, 34]. Based on the TRAPD (Translation, Review, Adjudication, Pretesting, and Documentation) model, two independent and external translators will each provide drafts of a common forward and backward translation, which will subsequently be discussed and reviewed by an expanded group of bilingual experts. The approved versions will then be pretested. The entire procedure will be repeated, if necessary. This multi-step team translation approach not only ensures the reliable detection and elimination of problems of equivalence between the different questionnaire versions [35], but also takes into account the cultural specifics of the respective language. All other study materials (e.g. informed consent, brochure for the CG) will be translated by bilingual experts as well.

**Table 1:** Overview of data to be collected for the evaluation of a self-help app for traumatized Syrian refugees

Construct (Assessment)	Screening	Baseline (T0)	Follow–up (T1 / T2)	Arabic translation necessary
Sociodemographics				
Age	x			yes
Sex	x			yes
Education	x			yes
Marital status	x			yes
Living situation	x			yes
Residence status	x			yes
Occupational status	x			yes
Monthly income	x			yes
Escape-related information				
Home country	x			yes
Date of escapes’ beginning	x			yes
Duration of stay in Germany	x			yes
Means of escape	x			yes
Strength of bonding to homeland / current residence	x			yes
Future plans	x			yes
Satisfaction with current situation	x			yes
Additional inclusion criteria				
Smartphone availability, no current psychiatric treatment and/or no psychopharmaceutic medication, no pregnancy	x			yes
Suicidal tendencies (DSI-SS)	x	x	x	yes
Primary Outcome				
Post-traumatic stress disorder (PDS – 5)	x	x	x	yes
Secondary Outcomes				
Depression (PHQ-9)	x	x	x	no
Anxiety (GAD-7)		x	x	no
Somatization (PHQ-15)		x	x	no
Quality of life (EQ-5D-5 L)		x	x	no
General self-efficacy (GSE)		x	x	yes
Self-stigmatization (SSMIS-SF)		x	x	yes
Ambiguous loss and prolonged grief		x	x	yes
Social network (LSNS)		x	x	yes
Social support (ESSI)		x	x	yes
Post-traumatic growth (PGI)		x	x	yes
Resilience (RS-13)	x			yes
Religious beliefs (Z-7)		x	x	yes
Healthcare resource use		x	only T2	yes
App usability (SUS)^a^		x	only T1	yes
User acceptance (TAM 3)^a^		x	only T1	yes

Note: ^a^to be assessed only in the intervention group

*DSI-SS* Depressive Symptomatology Index – Suicidality Scale [47], *PDS-5* Posttraumatic Diagnostic Scale for DSM-5 [30], *PHQ-9* Patient Health Questionnaire [31], *GAD* General Anxiety Disorder [48], *EQ-5D-5L* Euroqol Five Dimensions Questionnaire [49], *GSE* General Self-efficacy [50], *SSMIS-SF* Self-stigma of Mental Illness Scale – Short Form [51], *LSNS* Lubben Social Network Scale [52], *ESSI* Enriched Social Support Inventory [53], *PGI* Posttraumatic Growth Inventory [54], *RS-13* Resilience Scale [55], *Z-7* Zentralitätsskala [56], *SUS* System Usability Scale [36], *TAM-3* Technology Acceptance Model [37]

Ambiguous loss and prolonged grief - questions have been adapted from the German version of the Complicated Grief Inventory (ICG-D; [57]); Healthcare resource use – questionnaire was adapted from previous investigations and is available from the authors upon request [58]

We hypothesize that the app intervention reduces posttraumatic stress symptomatology (primary outcome), quantified using the Posttraumatic Diagnostic Scale for DSM-5 (PDS-5 [30]). The PDS-5 is a well-established self-report measure based on the diagnostic criteria of the DSM-5, which allows the assessment of posttraumatic stress symptom severity.

Regarding secondary outcomes, we moreover expect the intervention to reduce symptoms of depression, anxiety, and somatization. Positive intervention-related effects are also expected regarding quality of life, self-efficacy, perceived stigmatization, social support, ambiguous loss, and posttraumatic growth.

Use of resources in terms of health demands will be assessed using a cost book (bottom-up approach). Furthermore, we aim to gather information on user acceptance and usability of the self-help app using the System Usability Scale (SUS) [36] and Technology Acceptance Model (TAM3 [37]), respectively.

Last, meta-data stored in the app’s log-files will provide quantitative information about the use of the app, e.g. number of days used, number of worked-through modules, or operating time.

#### Timeline

**Fig. 3:**
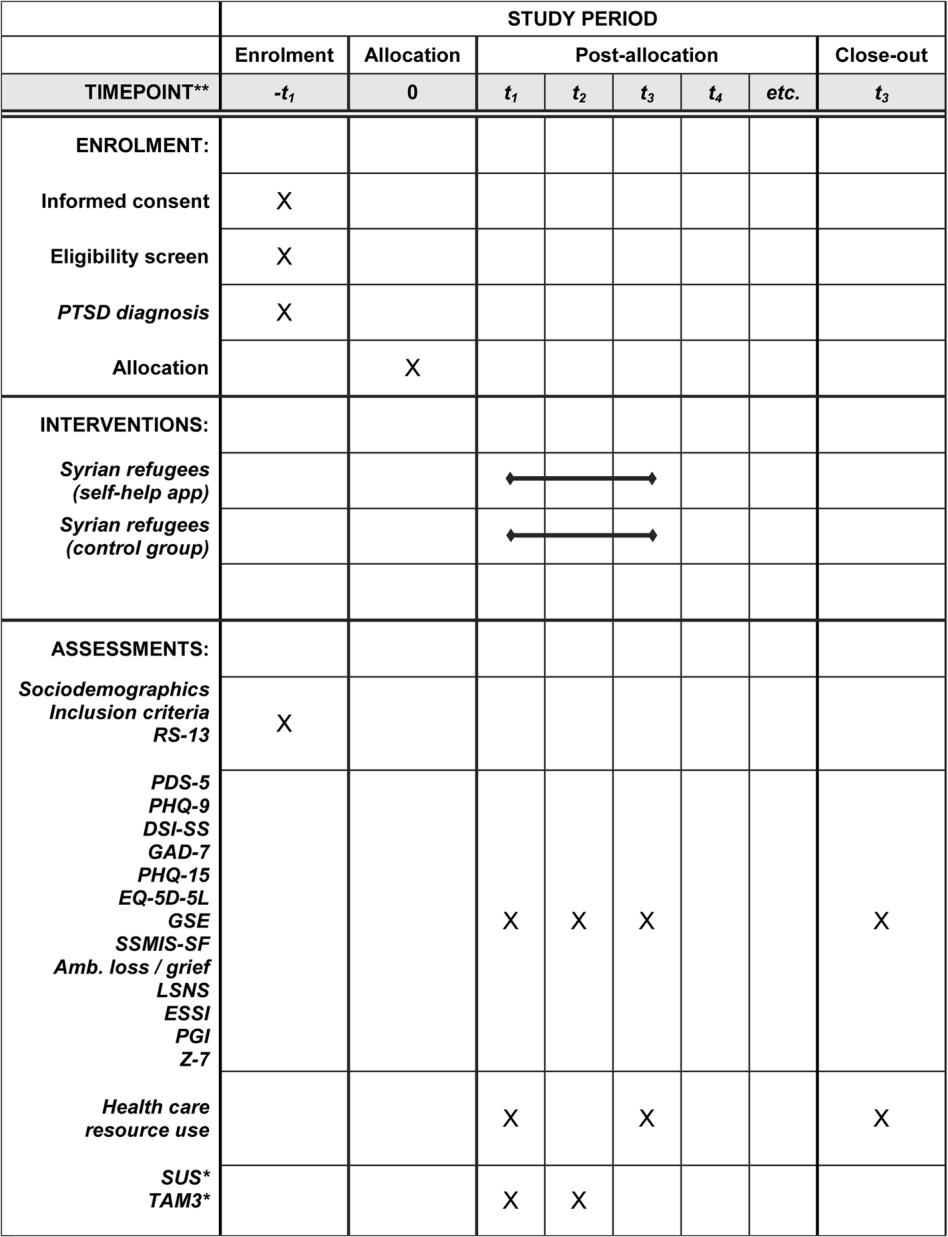
Schedule of enrollment, interventions and assessments. Completed SPIRIT 2013 figure of recommended content of enrolment, interventions, and assessments. Abbreviations: t1 = baseline; t2 = 4 weeks after baseline (post intervention); t3 = 16 weeks after baseline (follow-up). *assessed in intervention group only

After positive screening, study participants will be enrolled. Measurements are scheduled directly before the intervention (baseline, T0/pre), immediately after the intervention (T1/post, one month later), and three month after the intervention (T2/follow-up) to test for medium-termed treatment effects. A detailed overview of the schedule of enrolment, interventions and assessments is given in Fig. 3.

#### Sample size

Anticipated sample size was calculated based on recent research evaluating the efficacy of telemedical-based treatment of posttraumatic stress symptoms [20, 21, 38]. Given a moderate between-group effect at T1 (Cohen’s *d* = 0.5), a significance level of *α* = 0.05 and a statistical power of 1-*β* = 0.90, optimal sample size for estimating a significant treatment effect is *n* = 140. Expecting a drop-out rate of approximately 40% (e.g. due to change in residence status), *n* = 94 participants have to be included additionally, yielding an ideal baseline sample of *n* = 234 participants at T0 [39].

#### Recruitment

A multi-strategic approach to recruit Syrian refugees living in the urban area of Leipzig, German, will be targeted. This includes using established contacts as well as building new contacts with facilities frequented by Syrian refugees (e.g. refugee related clubs, associations and organizations, psychosocial centers, information centers, language schools, community colleges, universities, registration authorities, hospitals, clinics, primary care practices, psychotherapeutic practices, etc.). These facilities act as multipliers to reach out to potential study participants. Likewise, participants will be recruited from local reception centers and other forms of collective accommodation centers. We will use social media, instant messaging and online tools to spread information about the study. The local media will be involved. Bilingual flyers and posters will be displayed all across the city in appropriate public spots. During the recruitment phase, we will have an information booth at local events that might attract Syrian refugees. Importantly, since Syrian refugees are well connected with each other, we aim to reach additional participants of the Syrian community by using a snowball sampling approach, e.g. we ask participants to spread information about the study within their personal social network [29].

#### Allocation and randomization

Participants identified as eligible during screening will subsequently be randomly assigned to the IG or CG. Group assignment will follow a 1:1 ratio utilizing randomized permuted blocks of six, stratified by age and sex. This procedure ensures both balance in sample size across groups and control of important covariates. An external, independent statistician will generate the randomization block lists with a respective computer program (“blockrand”-package [40] written for R [41]). The different lists will be coded by this person so that the study coordinator, responsible for individual group allocation, remains blind to the lists’ strata identity.

#### Blinding

Besides randomization, the data analysts, who will conduct the primary analysis concerning the hypothesized group differences (IG vs. CG) in our primary and secondary outcome measures (see above), will be blind to group assignment.

#### Data collection

All assessments will be carried out in form of structured face-to-face interviews led by trained native Arabic speaking study nurses. The structured interviews will be present in form of paper-and-pencil assessments that the study nurses will complete to collect data on sociodemographics, primary and secondary outcomes using standardized questionnaires, as detailed above. Additionally, data will be collected to monitor possible negative effects and reasons for dropout (see below).

Data on the app usage will be collected through meta-data log-files that store anonymous information about the number of login-times and worked-through modules.

#### Data management

Data entry will take place successively and timely after collection using a statistical package. However, a data completeness and consistency check will take place beforehand. In case of missing answers to items, reasons for missing information will be clarified and efforts undertaken to reach completion. De-identified data will be entered in a data mask which will be password-protected and stored locally, with access granted only to study personnel who have signed data protection wavers. Anonymous log-file data of app usage will be collected, stored and transferred using SSL-technology to ensure data encryption and protection. In fact, specific arrangements regarding data protection (e.g. separation of evaluation and program, no real names for program login) will be defined by a separate data protection concept.

#### Statistical analysis

All acquired data will be examined with respect to potential inconsistencies and missing values, which will be replaced using multiple imputation methods [42], if appropriate. To detect potential systematic biases, a drop-out analysis will be performed. Internal consistencies of the newly translated inventories will be calculated to evaluate their reliability. All subsequent analyses will be treated as intention-to-treat or completer-analyses. Data-analysts will be blinded for group allocation.

Changes in primary and secondary outcomes will be described using mean scores and standard deviations, among others. Short and medium termed treatment effects will statistically be inferred by using linear mixed effect models accounting for relevant covariates. Usability as well as user acceptance of the app will be evaluated using descriptive statistics. The significance level of all analyses will be set to *p* < 0.05.

To assess the potential health economic benefits, we will perform analyses of the (i) actual costs and (ii) the ICER of the intervention. Costs will be quantified by the frequency of how often physicians and therapists are consulted (including days spent in a hospital) and valuated using current standard costs [43]. The app development expenses will be treated as economically reasonable overhead costs and added accordingly. To assess the interventions’ economic efficiency, the ICER will be calculated as the ratio between the difference of the actual costs and the difference of the averaged treatment effects (indexed by either quality-adjusted life years or primary outcome scores) between IG and CG. Respective confidence intervals will be derived by non-parametric bootstrapping. To visualize the ICERs statistical uncertainty, cost-effectiveness-acceptance curves will be constructed, indicating the probability of an acceptable ICER in relation to willingness to pay for an effect. Alternatively, net benefit regressions [44] can be applied to estimate the respective group specific net benefits while accounting for potentially confounding variables such as age, sex, and education.

The results of the study will be reported according to the guidelines of the Consolidated Standards of Reporting Trials (CONSORT) statement [45].

### Monitoring

#### Data monitoring

A data monitoring committee (DMC) will not be established for several reasons. First, periodic inspection does not seem justified as trial duration is short (4 weeks). Second, overall risk associated with the self-help intervention is considered low, and therefore, the likelihood for the need to modify or discontinue the trial is considered insignificant. However, precautions have been taken to minimize the potential of any harm towards individual study participants, as detailed below.

#### Harms

Preventively, we will only enroll study participants without severe posttraumatic and/or depressive symptomatology and acute suicidality. The self-help program targets individuals with mild to moderate symptom severity. Despite these precautions, the occurrence of negative effects during the trial in individual cases cannot be entirely ruled out. Therefore, potential negative effects will be monitored through a standardized assessment procedure at follow-up 1 (post-intervention) and follow-up 2 (four months after baseline). Negative effects are defined as adverse events (AE) and severe adverse events (SAE). Specifically, AE comprise i) an increase in target symptoms (posttraumatic and depressive symptomatology, suicidality), ii) the occurrence of novel psychological or physical symptoms, and iii) any negative events, all of which may or may not be associated with trial participation, in both IG and CG. Participants will be questioned whether any AE is thought to emerge from trial participation. SAE are defined as severe negative events that occur during study participation and result in adverse reactions requiring some form of high intensity treatment (i.e., deliberate self-harm, suicide attempt, life-threatening events, non-elective or extended hospitalization, an event causing chronic or serve disability) or fatality, including suicide. SAE will be surveyed and reported regardless if they are deemed related to the trial or not. The principle investigator (SRH) will be immediately informed about the occurrence of any AE and SAE. Likewise, a consensus conference (incl. the principle investigator and clinical investigators) will be held immediately to decide on appropriate measures in response to AE and SAE. Moreover, data collected on AE and SAE will be analyzed and reported as part of the study outcomes.

Practically, during the trial, study participants of both groups (IG and CG) will be provided with detailed information regarding emergency hotlines and professional help, if needed, i.e., in case of unexpected symptom deterioration. The IG will have access to this information via an emergency button leading to a help menu within the app. The CG receives the same information as part of the psychoeducational reading material that is handed out at baseline. In addition, study participants of both groups will be encouraged to contact our study nurses if they need further support to find emergency help.

#### Auditing

Auditing will take place in form of independent reviews of the data collection across all assessment waves. Specifically, 5 % of the questionnaires at baseline, follow-up 1 and follow-up 2, respectively, will be randomly drawn and inspected regarding their degree of matching with the database input. Source data verification will be performed independently from investigators and study sponsors by commissioned, external statisticians.

#### Protocol amendments

In case of necessary protocol amendments, we will describe all relevant details/changes and associated rationales.

#### Dissemination policy

The results of the RCT will be published in international, peer-reviewed journals. Moreover, we plan to present our findings at international and national conferences and meetings relevant to the field. To reach an even broader publicity, we aim to cooperate with the largest health insurance provider in Germany (AOK - Federal Association of Local Health Insurance Funds), national institutions concerned with migration and integration as well as stakeholders/protagonists of refugee care centers. We will additionally arrange several information events for experts from the field of psychiatry and psychology as well as for trauma-specific experts and representatives of psychosocial counselling centers. Lastly, we aim to share our results via public media channels. We aim to make the app freely available to everyone.

## Discussion

We aim to develop and evaluate an interactive self-help app in Arabic for Syrian refugees with symptoms of posttraumatic stress. We expect that the use of our self-help app will lower respective and associated comorbid symptoms, e.g. depression or anxiety, over a course of four weeks. Moreover, our evaluation will consider the intervention’s cost-effectiveness as well as aspects regarding usability and user acceptance. We plan to make the app publicly available. Hence, we hope to provide a flexible low-threshold treatment for Syrian refugees, who suffer from the consequences of traumatization, but who may lack access to an appropriate treatment. If proven to be efficient, the self-help app could fill an important treatment gap in health services provision to a large group in need, and thus, could significantly improve the psychosocial care for Syrian refugees in Germany. Moreover, after implementation in public health, applicability may not only be restricted to Syrian refugees, but the app can be helpful for refugees from other Arabic-speaking countries with similar cultural backgrounds.

However, the realization of the project might be subject to some risks that have to be considered. Regarding the app development, it is important to adequately adapt the content to culture-sensitive aspects (e.g. disease concepts, the specific situation of refugees in Germany). Therefore, insights obtained from interviews with respective focus groups will be integrated. While the translation of the app materials and research inventories need to be culturally sensitive, it should not lose the necessary precision. In order to optimally balance these demands, we will cooperate with qualified translators, bilingual and native Arabic speaking experts, who will translate and review all materials within the standard forward and backward translation process (TRAPD-Model). Lastly, difficulties might occur with respect to recruitment and adherence of participants, e.g. due to contact barriers, dropout, or imminent deportation. We therefore aim for a multi-strategic recruitment approach. In addition, we applied a high dropout rate (40%) for sample size calculation.

Although a recent meta-analysis demonstrated the efficacy of internet-based interventions for posttraumatic stress [20, 21], the associated advantages and potentials remain unused in Germany. The same seems to be true with respect to mHealth approaches. Although, there are plenty of self-help apps available, most of them have not been validated yet [46], and might not meet the specific needs of traumatized Syrian refugees. Thus, we will develop and evaluate an interactive self-help app in Arabic language, which will prioritize the special needs of Syrian refugees in Germany. We expect that our results will extend current knowledge regarding the efficacy of mobile health interventions in the field of psychosocial care for traumatized Syrian refugees in Germany. The overall goal is to provide the app to the general public for free as a low-threshold add-on to routine care, which currently lacks adequate treatment options for refugees with posttraumatic stress.

## Abbreviations

AE: Adverse event
App: Application
AWMF: German Association of the Scientific Medical Societies
CBT: Cognitive-behavioral therapy
CG: Control group
DMC: Data monitoring committee
DSM-5: Diagnostic and Statistical Manual of Mental Disorders, 5th Edition
ICER: Incremental cost-effectiveness ratio
IG: Intervention group
mHealth: Mobile health
PDS-5: Posttraumatic Diagnostic Scale for DSM-5
PHQ-9: Patient Health Questionnaire
PTSD: Posttraumatic stress disorder
RCT: Randomized controlled trial
SAE: Severe adverse event
